# Alternative to Chemotherapy—The Unmet Demand against Leishmaniasis

**DOI:** 10.3389/fimmu.2017.01779

**Published:** 2017-12-21

**Authors:** Nicky Didwania, Md. Shadab, Abdus Sabur, Nahid Ali

**Affiliations:** ^1^Infectious Diseases and Immunology Division, CSIR-Indian Institute of Chemical Biology, Kolkata, India

**Keywords:** leishmaniasis, vaccine, immunotherapy, chemotherapy, immunology

## Abstract

Leishmaniasis is a neglected protozoan disease that mainly affects the tropical as well as subtropical countries of the world. The primary option to control the disease still relies on chemotherapy. However, a hindrance to treatments owing to the emergence of drug-resistant parasites, enormous side effects of the drugs, their high cost, and requirement of long course hospitalization has added to the existing problems of leishmaniasis containment program. This review highlights the prospects of immunotherapy and/or immunochemotherapy to address the limitations for current treatment measures for leishmaniasis. In addition to the progress in alternate therapeutic strategies, the possibility and advances in developing preventive measures against the disease have been pointed. The review highlights our recent understandings of the protective immunology that can be exploited to develop an effective vaccine against leishmaniasis. Moreover, an update on the approaches that have evolved over the recent years are predominantly focused to overcome the current challenges in developing immunotherapeutic as well as prophylactic antileishmanial vaccines is discussed.

## Introduction

*Leishmania*, a protozoan parasite, causes a complex form of disease called leishmaniasis. This disease is prevalent in 98 countries with major occurrence in the developing world (India, Bangladesh, Nepal, Ethiopia, Sudan, and Brazil) (1). Approximately 20,000–30,000 deaths and 0.7–1 million new cases of leishmaniasis occur per annum. The clinical forms include visceral (the most serious form of the disease, also known as kala-azar), cutaneous and mucocutaneous leishmaniasis (ML) (WHO, 2017). *Leishmania* parasites are transmitted in the mammalian host by infected sandfly. These flies mostly belong to the genus *Phlebotomus* and *Lutzomyia* (2). The control of leishmaniasis mainly relies on chemotherapy while other measures include sleeping under nets treated with insecticide, and spraying insecticides to kill sandflies. The current therapy against leishmaniasis depends on the use of the drugs such as pentavalent antimonials and amphotericin B. Other treatment options include miltefosine and paromomycin. The lacunae suffered by these drugs are inherent toxicity and requirement of long-term treatment. In addition, the expansion of human immunodeficiency virus (HIV) has influenced the epidemiology of the leishmaniasis. 35 out of the 70 countries, endemic for visceral leishmaniasis (VL), have documented cases of *Leishmania*–HIV coinfection (3). One of the unfavorable complications amalgamated with HIV coinfection is that it lowers the plausibility of a therapeutic response to treatment against *Leishmania infantum* and it also greatly boosts the possibility of a relapse (4). Furthermore, the problem of high cost and the emergence of resistant parasites to these drugs have frustrated the situation of leishmaniasis control (5). These issues necessitate alternative to chemotherapy like developing new non-toxic antileishmanials or different interventions like prophylactic and therapeutic vaccine. Interestingly, development of resistance to reinfection in individuals cured of *Leishmania* encourages the feasibility of protective vaccine. Moreover, application of cytokines and immunomodulators as immunotherapeutic agents that can direct curative immune response provides promising approach to immunotherapy against leishmaniasis. Since the sequencing of complete *Leishmania* genome has been achieved, advancement toward understanding the disease pathogenesis along with its defense by the host has paved new opportunities in the way for *Leishmania* vaccine and immunotherapy research. This review will discuss the development in the field of prophylactic and therapeutic vaccine and the challenges encountered as alternatives to chemotherapy against leishmaniasis.

## Immunobiology of Leishmaniasis: Insights of the Disease

The basic necessity for developing any form of interventions against the disease is the better understanding of the host–pathogen interaction. The characteristic that allows the parasite to establish chronic infection lies in its ability to dampen as well as evade both the innate and adaptive machinery of the host’s immune system. The major innate immune cells that play a significant role in defense against *Leishmania* are neutrophils, macrophages, and dendritic cells (DCs). When the female sandfly sucks blood meal from the vertebrate hosts, flagellate metacyclic forms of *Leishmania* are delivered along with sandfly salivary ingredients into the skin of the hosts (6). Initially, the promastigotes are taken up by the neutrophils at the site of infection. Following apoptosis in these infected neutrophils, the released parasites infect neighboring macrophages (7, 8). These macrophages are recruited by the chemotactic properties of the proteophosphoglycans, delivered to the infection site by the vector at the time of its blood meal (9, 10). Binding of *Leishmania* parasites to C3b accelerates phagocytosis after which promastigotes get converted to the amastigote form (11). Following phagocytosis in macrophages, establishment of infection is determined by several survival strategies of the parasites, most prominent of which is the modulation and attenuation of immune responses. *Leishmania* parasite suppresses the release of Th1 associated cytokines like interleukin (IL)-12 from these cells. This, in turn, restrains DCs to present the parasite-specific antigens to the T cells. Thus preventing the activation of the acquired immunity, this is very crucial for the containment of the disease (12, 13). Influence of Th1/Th2 cytokine has been observed to vary in the progression of disease in VL compared with cutaneous leishmaniasis (CL). Although the classical Th1/Th2 paradigm of resistance/susceptibility appears to be valid during CL, a mixed Th1/Th2 response is required for disease control during VL (14). However, it is yet to establish a clear Th1/Th2 paradigm for curative and preventive response against both CL and VL. Moreover, for ML, the disease manifestation is largely due to inflammatory response than due to parasite burden. Therefore, conventional Th1/Th2 paradigm does not apply to ML. It has been found that Treg (CD4^+^CD25^+^ regulatory T cells) as well as Th17 (other subsets of T cells) cells, play a significant role in disease outcome in both CL and VL, their role in ML is much more complicated. Studies with *Leishmania major* and *L. infantum* have shown a protective role of IL-17 as well as IL-22 (Th17 cytokines) against intracellular parasites (15, 16). Recently it was shown that when recombinant IL-17 or IL-23 was administered to mice it caused a considerable containment of parasite load in infected organs with significant production of factors such as IFN-γ, nitric oxide, etc. Thus, this study demonstrated the association of Th17-based cytokines in providing protection against the disease (17). As an important constituent of the immune system, Treg cells are known to regulate immune response of other cells. These cells were observed to be present in human cutaneous lesions (18). Increased expression of lesional FoxP3 and IL-10 during progressive *L. major* infection in a murine model and similarly during *Leishmania braziliensis* infection in human patients suggest the disease-promoting role of these regulatory cytokines (19). The preliminary data suggest that despite Th1 polarization production of IL-10 and Treg cells is associated with delayed healing of CL (20). Apart from T-cell subsets (Treg and Th17) other than conventional T cells, the role of innate immune response has been essentially linked to disease outcome. In fact, engagement of the macrophage toll-like receptors (TLRs) by the parasites has not only shown to improve phagocytosis but also lead to the killing of the parasites due to triggering of NF-κB transactivation and concomitant production of the downstream mediators including pro-inflammatory cytokines. For instance, TLR9 activation has been found to be beneficial for the host against these parasites. But this situation may not be true for all the TLRs. Lipophosphoglycan, a TLR2 agonist has been shown to have antagonizing effect on TLR9 mediated signal cascade in host macrophage, which in turn facilitates parasite survival (21, 22). Studies showed that treatment with TLR4 and TLR9 agonists decreased the disease severity following challenge infection with *L. major* in BALB/c mice (23). However, in human VL, comparison of mRNA expression levels between pretreatment and posttreatment splenic aspirate samples showed considerably more TLR2 and TLR4 expression but no change in TLR9 expression during *Leishmania donovani* infection (24). Despite advances, achieving a comprehensive and clear picture of the immunobiology of leishmaniasis is still required to develop effective interventions such as type-specific vaccine and immunotherapy for leishmaniasis.

## What Necessitates a Vaccine?

Chemotherapy is the key intervention to control leishmaniasis. The existing drugs for the treatment of leishmaniasis in the market are pentavalent antimonials, amphotericin B, miltefosine, paromomycin, and amphotericin B in liposomal forms. But the major setback of these drugs include toxicity, cost, route of injection, treatment duration, and the predominant one being blooming of drug-resistant parasites (5). Pentavalent antimonials are the first line course of medication for leishmaniasis worldwide except Indian subcontinent. In India, about 90% of all infections are resistant to pentavalent antimonials (25). Accordingly, amphotericin B has been used as the leading drug to treat patients infected with *Leishmania*. But high toxicity and reports of drug-resistant parasites have narrowed down their use (26). Miltefosine, an oral medication, came up with promising results in the beginning but there is increasing occurrence of relapses in cases prescribed with this drug (27–29). Lately, in a multicentric clinical trial, it was found that Ambisome (liposomal amphotericin B) was effective enough in a single-dose treatment with a lesser degree of toxicity compared with mainstream treatments (30). However, this line of medication protocol raises the possibility of advancement in the development of drug-resistant parasites. Consequently, there is a development of combinatorial drug therapy for use in endemic regions (31, 32). Nevertheless, mouse model studies imply that even the combinatorial drug treatment can develop drug resistance *L. donovani* (33). Regardless of the advances in antileishmanial chemotherapeutics, it is implausible that chemotherapy solely will facilitate disease eradication. Since leishmaniasis is predominantly a disease of the poverty-stricken community, chemotherapy proves inadequate and less acceptable. This socioeconomic concern calls for a preventive and/or immunotherapeutic alternative to chemotherapy. Hence there is an imperative need for an effective prophylactic and therapeutic vaccine if enduring ambition of managing and eliminating this disease are to be accomplished.

## Prospects of Vaccine Development

Even though the global share of leishmaniasis is limited to selective parts of the world, the number of individuals being affected and are at risk is noteworthy. The ongoing scope to deal with this concern includes vector control, development of technologies for easy and quick diagnosis, refinement of drugs for improved treatment and developing vaccine approaches both prophylactically and therapeutically.

### Prophylactic Vaccine Approaches

Admittedly, at present, there is no human administrable vaccine against leishmaniasis. However, development is in progress. At present, the vaccine designing approaches (Table 1) are broadly grouped as follows: (a) genetically manipulated live vaccines; (b) preparation of whole killed parasites or their fractions; and (c) vaccines based on defined molecules, which include recombinant protein vaccines and/or DNA vaccines as single or multiantigen combinations.

**Table 1:** Prophylactic vaccine approaches against leishmaniasis.

Description of antigen	Vaccine approaches	Animal model	Adjuvant	Reference
p27 gene knockout *Leishmania donovani* parasites	Live attenuated	Mouse	–	(34)
Centrin-deficient parasites of *L. donovani*	Live attenuated	Dogs	–	(35)
Live mutants of *Leishmania* lacking genes like dihydrofolate reductase, biopterin reductase, and cysteine proteases (CPs)	Live attenuated	Mouse	–	(36)
Ascorbic acid-deleted live mutants of *L. donovani*	Live attenuated	Mouse	–	(37)
Merthiolated sound-disrupted *Leishmania braziliensis*	Killed vaccine	Dogs	BCG	(38, 39)
Killed *L. braziliensis*	Killed vaccine	Dogs	Saponin	(40, 41)
KBMA *Leishmania infantum chagasi*	Killed vaccine	Mouse	–	(42)
Soluble leishmanial antigens of *L. donovani* promastigotes	Fractioned vaccine	Mouse	MPL-TDM	(43)
115 kDa soluble serine protease	Fractioned vaccine	Mouse	IL-12	(44)
*L. braziliensis* promastigote proteins	Fractioned vaccine	Dogs	Saponin	(45)
Leishmune (purified *L. donovani* fraction FML)	Fractioned vaccine	Dogs	Saponin	(46)
*L. donovani* p45 (rLdp45)	Recombinant protein	Hamster and human	–	(47)
*Leishmania tarentolae* expressing *L. donovani* A2 antigen along with CPs [CPA and CPB without its unusual C-terminal extension (CPB^−CTE^)]	Recombinant vaccine and DNA vaccine	Mouse, dogs	–	(48–50)
*Leishmania major* ribosomal protein L3 or L5	Recombinant protein	Mouse	CpG-ODN	(51)
Recombinant *L. tarentolae* stably expressing CP (CPA and CPB) with PpSP15 (protein from the sandfly *Phlebotomus papatasi*) DNA	Recombinant vaccine	Mouse	–	(52)
*Lactobacillus lactis* expressing LACK and mouse IL-12	Recombinant vaccine	Mouse	–	(53)
Leish-Tec (*L. donovani* amastigote-specific protein A2)	Recombinant protein	Dogs	Saponin	(54)
*L. infantum* acidic ribosomal P0	Recombinant protein	Hamsters	–	(55)
Recombinant *L. tarentolae* secreting PpSP15	Recombinant vaccine	Mouse	CPG-ODN	(56)
Cocktail of *L. donovani* CPs types I, II, and III	Recombinant protein cocktail vaccine	Hamsters	MPL-TDM	(57)
Cocktail of rCDV-LACK, rCDV-TSA, and rCDV-LmSTI1	Recombinant protein cocktail vaccine	Dogs	–	(58)
Recombinant *L. donovani* protein disulfide isomerase	DNA vaccine	Hamsters and human	–	(59)
*L. major* TSA	DNA vaccine	Mouse	Aluminum phosphate	(60)
*Leishmania amazonensis* iron superoxide dismutase	DNA vaccine	Mouse	–	(61)
Cocktail of *L. major* CPs type I, II, and III	Cocktail DNA vaccine	Mouse	–	(62)
T-cell epitope of KMP11, CPA, CPB, EF1α, and TSA (LEISHDNAVAX)	Multiantigenic T-cell epitope fusion DNA vaccine	Mouse	–	(63)
*L. donovani* surface GP63	Recombinant protein, DNA vaccine, and T-cell epitope peptide vaccine	Mouse, human	MPL-TDM and CPG-ODN	(64–66)
Chimeric peptides containing HLA-restricted epitopes from three immunogenic *L. infantum* proteins (CPA, histone H1, and KMP 11)	Peptide vaccine	Transgenic mouse	Poly(lactic-*co*-glycolic) acid nanoparticles and/or MPL-A	(67)
Chimeric peptides containing HLA-A2 restricted epitopes from six immunogenic *L. major* proteins (CPB, CPC, LmsTI1, TSA, LeIF, and LPG-3)	Peptide vaccine	–	–	(68)

*KBMA, killed but metabolically active; FML, fructose mannose ligand; CPA, cysteine protease A; CPB, cysteine protease B; rCDV, recombinant canine distemper virus; LACK, Leishmania homolog for receptors of activated C kinase receptor; TSA, thiol-specific antioxidant; LmTSI1/LmsTI1, L. major homolog of eukaryotic stress-inducible protein 1; KMP11, kinetoplastid membrane protein 11; EF1α, elongation factor 1-alpha; LeIF, Leishmania elongation initiation factor; MPL-A, monophosphoryl lipid A; MPL-TDM, monophosphoryl lipid A-trehalose dicorynomycolate; ODN, oligodeoxynucleotide; GP63, glycoprotein 63; HLA, human leukocyte antigen; CPC, cysteine protease C; LPG-3 lipophosphoglycan biosynthetic protein; CPG-ODN, CpG-oligo-deoxy-nucleotides; IL, interleukin*.

#### Live but Attenuated *Leishmania* Parasites As Vaccine

Vaccination with live parasites has always been an appealing approach as it mimics the natural infection. An effective vaccine comprising of live and virulent parasites termed as leishmanization has existed in the past. However, development of nonhealing lesions in some individuals led to the issue of questionable safety of this approach and it was discontinued in most of the countries except Uzbekistan (6). More recently, the aptness in expertise to edit the *Leishmania* genome to design genetically engineered parasites by deleting essential virulent genes rejuvenates the dormant utility of live attenuated *Leishmania* vaccine. Recently, in an approach to test the feasibility of live attenuated vaccine, researchers attenuated live *L. donovani* parasites by deleting centrin which on immunization exhibited protective immunity against *L. infantum* infection in dogs (35). In another study, mutant *L. donovani* obtained by deleting gene of ascorbic acid was shown to confer long-term protection against VL (37). Similarly, attenuated *Leishmania* parasites derived by deleting promising genes including cysteine proteases (CPs), biopterin reductase, and dihydrofolate reductase manifested significant protection in vaccinated mice against challenge infection with virulent parasites (36, 69). Studies on p27 gene knocked out live attenuated *L. donovani* parasites in BALB/c shows induction of long-term protective immunity (34). Recently, growth arrested live attenuated amastigotes of *Leishmania* have been explored as an encouraging technique for vaccine development against VL (35). Even though genetically attenuated live vaccines have been shown to be efficacious in experimental model, the safety of these mutants following mass vaccination cannot be affirmed because in immunocompromised individuals and HIV-positive persons the possibility of vaccine-induced leishmaniasis still remains.

#### Killed or Avirulent *Leishmania* Parasites As Vaccine

Pioneering work of the late 1930s by Brazilian scientists has shown the therapeutic and prophylactic efficacy of killed parasite vaccines against CL and VL. From then, it was realized that if the biochemical composition including antigenicity of these killed *Leishmania* parasites remains unperturbed it could be used as a promising vaccine candidate. In 1990s, Mayrink and coworkers, using merthiolated sound-disrupted *L. braziliensis* and BCG vaccine, showed 90% protection in phase I and II clinical trial against experimental canine VL challenged with *Leishmania chagasi*. However, in a well-designed field Phase III trial, this vaccine formulation failed to show any significant difference in dogs as compared with placebos (38, 39). The authors speculated that the difference between the artificial and the natural challenge could account for this failure in the field assay. Giunchetti et al. experimented with killed *L. braziliensis* vaccine along with saponin and/or sandfly saliva extract and got some quite significant results (40, 41). However, these speculations were little hindered because the whole-parasite based vaccine failed to confer significant protection to humans against leishmaniasis (70). Recently, an attempt was made to improve the efficacy of whole-cell vaccination. For this first promastigote of *L. chagasi* was exposed to low dose of UV radiation to generate *Leishmania* organism termed killed but metabolically active. Further using this processed cells in combination with amotosalen, S-59; a psoralen compound, this vaccine conjugate was shown to have promising results comparable to vaccination with the virulent live organisms (42). However, to develop vaccines out of the whole cell is still a challenge that limits its widespread use. Therefore, despite being a safe and dependable option, killed parasite vaccines demand further in-depth investigation for a stable alternative.

#### Purified Fractions of Leishmanial Lysate

Purified fractions, as well as subfractions, of the *Leishmania* parasites have shown significant immunoprotective profile when used in several vaccine models. For example, cationic liposomes encapsulating soluble antigens isolated from *L. donovani* promastigotes (SLA) when mixed with the adjuvant [monophosphoryl lipid A-trehalose dicorynomycolate (MPL-TDM)] and given subcutaneously to BALB/c mice conferred long-term protection against experimental VL (43). Similarly, fructose mannose ligand (FML), isolated from *L. donovani*, when used in combination with saponin (adjuvant) conferred significant protection against canine VL (46). Moreover, the vaccine not only provides a promising tool to prevent canine VL but is also advantageous in controlling transmission of zoonotic VL (71). Recently, the LiESP/QA-21 vaccine was licensed for commercialization under the name of CaniLeish^®^ in Europe. It is composed of purified excreted-secreted proteins of *L. infantum* (LiESP) adjuvanted with QA-21 (saponin) (72). However, problems associated with purification, as well as the large-scale production of these fractioned vaccines, are some of the limiting factors in their extensive use. Therefore, development of alternatives having vaccine potential including recombinant proteins, polyproteins, DNA vaccines are in the process as discussed in the following sections.

#### Immunogenic Recombinant Antigen Based Vaccine

The advent of the recombinant DNA technology has boosted enormously the vaccine development program against leishmaniasis. It has allowed generating leishmanial recombinant proteins as desired. These proteins owing to their high purity and yield provide an advantage of developing promising vaccine candidates. For example, recombinant GP63 expressed in bacteria was used as encapsulated form in cationic liposomes to immunize BALB/c mice in combination with TLR4 agonist-MPL-TDM. This combinatorial vaccine formulation was found to confer significant protection against murine VL through activation of both CD4 and CD8 T cell-mediated immune responses (64). Similarly, combination of CpG-oligo-deoxy-nucleotides, a known TLR9 agonist with recombinant ribosomal antigen L3 or L5 from *L. major*, improved the protection in two different murine models against homologous challenge infection (51). In addition, recently a new approach was taken to improve vaccine potential of the recombinant proteins. For instance, Katebi et al. genetically engineered (56) the non-pathogenic *Leishmania tarentolae* species to express and deliver a specific sandfly salivary antigen, PpSP15. Use of this recombinant *L. tarentolae*-PpSP15 with CpG conferred a significant protection against infection to *L. major*. Similarly, Saljoughian et al. developed (48) *L. tarentolae* that expresses *L. donovani* A2 antigen. When they used this genetically engineered cell along with CPs for immunization of BALB/c mice in a prime-boost manner, it showed significant protection against *L. infantum* challenge. Another group evaluated the immunogenicity and protective efficacy of *L. tarentolae* expressing a trifusion protein containing *L. donovani* A2 antigen along with CPs A and B without its unusual C-terminal extension (CPB^-CTE^) against *L. infantum* infectious challenge with prime-boost regimen in dogs. Vaccinated dogs showed higher levels of Th1 immune response with a strong DTH response and low parasite burden representing a promising candidate against canine VL (49). The strongest defensive efficacy in the mice models (C57BL/6 and BALB/c) against infection to *L. major* was observed by Zahedifard et al., when they primed these animals with PpSP15 DNA and boosted them with the combination of PpSP15 DNA and *L. tarentolae* (live) that was engineered to stably express genes for CPs (52). Recently, *Lactobacillus lactis* (alr-) strains are being used as an expression and delivery vehicles of biological compounds, such as cytokines and antigens, in mice and humans. In one such study, live *L. lactis* solely expressing the *Leishmania* antigen, *Leishmania* homolog for receptors of activated C kinase receptor (LACK) and mouse IL-12 was generated for orally immunizing BALB/c mice against *L. major* challenge. Immunization with the *L. lactis* expressing both LACK and IL-12 in secretory form induced LACK-specific Th1 immune response demonstrating the use of *L. lactis* as a live oral vaccine against leishmaniasis (53).

After a lot of effort in developing a vaccine against leishmaniasis, Leish-Tec, an amastigote-specific A2 recombinant protein vaccine against canine VL, is now commercially available in Brazil. Leish-Tec^®^ was found to be immunogenic in different breeds of canine population (54). Despite that these above vaccine formulations exhibited significant protection against various *Leishmania* models, scientists believe that a cocktail of different conserved antigens could provide better protection. In lieu of that a cocktail of recombinant canine distemper virus (rCDV)-LACK, rCDV-thiol-specific antioxidant (TSA), and rCDV-LmSTI1 was used to immunize dogs. This vaccine formulation provided significant protection to dogs against parasite challenge (58). Further, the use of cocktails of CPs in a hamster model of VL was found to be more effective when encapsulated in a liposome and delivered along with MPL-TDM (57). It is challenging to ensure persistent delivery of protein based antigens in intracellular milieu as a mimic of pathogenesis, therefore alternatively DNA-based vaccination strategy for cell-mediated immunity against leishmaniasis is sought to be explored.

#### DNA-Based Vaccines

DNA-based vaccines use bacterial plasmids, which are genetically engineered to encode antigens of interest. The advantage of these vaccines compared with the conventional live virus or protein subunit vaccines is that these are flexible, can be manufactured rapidly, are cost effective, and are able to induce cellular immunity (73). Immunization with DNA vaccine for expression of iron superoxide dismutase from *Leishmania amazonensis* is shown to protect BALB/c mice partially against challenge with the parasite (61). Tabatabaie et al. (60), however, showed that in addition with aluminum phosphate, TSA-based DNA vaccine confers significant protection against *L. major* infection in murine model. Shahbazi et al. did immunological comparison of electroporation and cationic solid–lipid nanoparticle delivery systems to administer a trifusion DNA vaccine A2-CPA-CPB^-CTE^ in dogs demonstrating both the systems as equally efficient vaccine delivery systems against canine VL (50). Interestingly, efficacy of CPs cocktail DNA vaccine was found to be enhanced following delivery in cationic lipid nanoparticles against murine model of CL exhibiting robust protective T-cell response (62). Besides these cocktail and fusion DNA vaccines, multiantigenic T-cell epitope-enriched DNA vaccine, LEISHDNAVAX has also developed showing significant protection. Its preclinical safety and tolerability studies have shown promising results. Moreover, distribution of the DNA vectors was systemic with no accumulation upon repeated injections. These results prompted initiation of clinical trials with the aim to use it for preventive as well as therapeutic applications (63). Heterologous immunization with DNA vaccine for priming followed by protein boosters using model antigen gp63 with CpG was shown to induce strong protective immune responses in mice. Moreover, it confers a long-term immunity to fight against the intracellular pathogens. The findings indicate that DNA-prime/protein-boost vaccination modality is superior to other possible combinations (65). Overall, these observations suggest that DNA vaccines are promising alternatives to conventional protein vaccines for controlling leishmaniasis. However, though DNA vaccines have proven their efficacy in animal models, they are strictly regulated for human use. Therefore, to potentially exploit DNA vaccines for the human use further effort are needed to make DNA vaccines safer avoiding autoimmune and cancer-related adverse effects.

#### Peptide-Based Vaccines

The peptide-based vaccine designing combines the benefits of a computational prediction with defined experimental validation to identify immunogenic epitopes within protein antigens. The boon of peptide-based vaccine design relies on their capability to trigger immune response solely dedicated to relevant epitopes overriding other irrelevant responses or unwanted side effects. Although the host defense mechanisms against leishmaniasis is not raveled completely, till date studies decipher the requirement of T cell-mediated response in controlling parasite infection. *In silico* approach of mining the proteome of parasites and analysis of the candidate antigens have helped in identifying both MHC class I- and class II-restricted T-cell epitope against *L. donovani* and *L. major*, which may serve as highly promiscuous peptides for developing subunit vaccine (74–76). Seyed et al. used *in silico* prediction to screen six *L. major* antigens for potential CD8^+^ T cell-activating epitopes presented by HLA-A*0201 (68). In another *in silico* approach, researchers refined 10 epitopes after screening thousands of epitopes derived from 8,000 proteins conserved in different *Leishmania* species. They tested the immunogenicity of these epitopes by stimulating the PBMCs of cured CL patients and only 50% of them were able to stimulate the proliferation of lymphocytes (77). Therefore, developing a plausible peptide-based vaccine needs to overcome quite a few considerable adversities. These include curbing the low immunogenicity and poor population coverage of individual peptide due to MHC restriction by combining multiple epitopes along with some immune response boosting adjuvant to target the adaptive immune response. Athanasiou et al. designed a chimeric peptide encapsulated in nanoparticle with monophosphoryl lipid A (MPL-A) or surface modification targeting TNF receptor II aimed to study their capability of stimulating the immunomodulatory properties of DCs. Chimeric peptide from three antigens incorporated in PLGA nanoparticle along with MPL-A were shown to induce maturation and activation of DCs imparting strong protective immunity against *L. infantum* infection in HLA A2.1 transgenic mice (67). Thus incorporation of multiple peptide-based epitopes in immunomodulatory delivery vehicles particles is a promising strategy for vaccine development against leishmaniasis.

### Immunotherapy

Immunotherapy comprises the use of biological and/or synthetic substances to modulate immune responses to that of cure. Strategies for immunotherapy include cytokine or chemokine treatment, antibody (Ab) blocking, immune modulation by vaccine antigens or adjuvants alone or in combination with chemotherapy. Different immunotherapeutic approaches against leishmaniasis have been listed in Table 2. In one such study, Castellano et al. administered antihuman monoclonal Ab in *L. braziliensis* SLA (soluble leishmanial antigens) stimulated cells from CL patients of endemic area with active or healed lesions to block IL-10 production, which showed decreased levels of IL-10, IL-4, and TNF-α in most of the patients except with active lesions. Moreover, there was limited alteration in production of an IFN-γ dependent chemokine, CXCL-10 (78). Another study examined the therapeutic efficacies of anti-glucocorticoid-induced TNF receptor-related protein and anti-IL-10R in *L. donovani*-infected C57BL/6 mice. Blocking IL-10 controlled parasite burden but combinatorial therapy using both the Abs could not suppress parasite proliferation in both liver and spleen even if there was low dose challenge. In addition, there was significant increase in TNF-α and IFN-γ in combinatorial therapy in comparison with single Abs (79). Abdossamadi et al. and Montakhab-Yeganeh et al. checked the suitability of transgenic *L. tarentolae* expressing leishmanial antigens such as human neutrophil peptide-1, and chemokines such as interferon-gamma-induced protein 10 or CXCL-10, respectively, as immunotherapeutic tools against *L. major*-infected BALB/c mice (80, 81). Both the approaches represented a promising immunotherapeutic strategy to improve treatment of CL. Immunomodulators, depending on their properties, can direct immune system response both negatively and positively. These immunomodulators can be exploited in a manner that can modulate immune response to control the infection. In an approach, researchers orally administered acetyl salicylic acid (ASA) as immunomodulator in *L. major*-infected BALB/c mice. ASA induced NO production, reduced proliferation of amastigote in macrophages, lesion size, and visceralization of parasites (82). Hoseini et al. used chitin and chitosan microparticles (MPs) as immunomodulators against *L. major* infection in BALB/c mice. Chitosan, acetylated form of chitin is a homopolymer extracted from shells of shrimp. Both the chitinous MPs showed reduced lesions and parasite load, induced cell proliferation and chitin but not chitosan induced TNF-α and IL-10 production (83). There are attempts to see the synergistic effect of various immunomodulators with chemotherapeutic agents as a combinatorial therapy against leishmaniasis. In one such attempt, studies were conducted to determine the lower dose effect of an antileishmanial drug, miltefosine, in combination with a single dose of tripalmytoil-cysteine (Pam3Cys) in BALB/c mice infected with *L. donovani*. The authors found this combination to be significantly effective in comparison with groups receiving Pam3Cys or miltefosine due to enhanced production of ROS, Th1 cytokines, and increase in phagocytosis index (84). Vaccines that can elicit cell-mediated immune response can be considered as potential candidate for immunotherapy, and there are various studies that are considering vaccine components with or without chemotherapy as immunotherapeutic tool. In one such study, Santos and coworkers found that Leishmune (saponin enriched) could significantly reduce the clinical symptoms and parasite load in the liver, spleen, bone marrow, and blood in seropositive and symptomatic dogs infected by *L. chagasi* (85). Borja-Cabrera et al. continued the study to compare the Leishmune (saponin enriched) with immunochemotherapy (saponin-enriched Leishmune in combination with allopurinol or AmB/allopurinol). They saw that immunochemotherapy cleared all the disease symptoms along with reduced infection and increased survival of the dogs infected with *L. donovani* (86). In an another canine VL study, glucantime treatment was compared with human trial candidate LEISH-F1 + monophosphoryl lipid A-stable emulsion (MPL-SE) in an Open Trial and a Blinded Trial. Glucantime alone failed to treat most of the cases whereas LEISH-F1 + MPL-SE was found to be effective not only for mild cases also but reduced the symptoms of severe canine VL as well (87). Recently, a study showed recombinant CP from *L. infantum chagasi* (rLdccys1) to be an effective immunotherapeutic agent against naturally infected dogs from Teresina, Piauí, a region of high incidence of VL in Brazil (88). Both murine and dogs along with human studies indicate immunotherapy to be a favorable alternative to conventional chemotherapy. However, there is lack of standardized immunotherapeutic protocols for use in treatment of leishmaniasis.

**Table 2:** Immunotherapeutic approaches against leishmaniasis.

Description of agent	Type of agent	Type of strain	Disease model	Reference
Acetyl salicylic acid	Immunomodulator	*Leishmania major*	Mouse	(82)
Pam3Cys and miltefosine	Chemoimmunotherapy	*Leishmania donovani*	Mouse	(84)
Cisplatin, 78 kDa antigen and MPL-A	Vaccines as immunotherapy	*L. donovani*	Mouse	(89)
rIL-2/anti-IL-2 Ab complex	Cytokine and immunomodulator	*Leishmania panamensis*	Mouse	(90)
Killed parasite (*L. donovani*), SSG, MPL-A, cisplatin, and antimony	Vaccines as chemoimmunotherapy	*L. donovani*	Mouse	(91)
Radio-attenuated *L. donovani*	Vaccines as immunotherapy	*L. donovani*	Mouse	(92)
Anti-hIL-10	Cytokine and immunomodulator	*Leishmania amazonensis*	Human	(78)
Liposomal amphotericin B and LEISHDNAVAX	Vaccines as chemoimmunotherapy	*L. donovani*	Mouse	(93)
Anti-IL-10R and anti-GITR	Cytokine and immunomodulator	*L. donovani*	Mouse	(79)
*Leishmania tarentolae* secreting HNP1	Vaccines as immunotherapy	*L. major*	Mouse	(80)
*L. tarentolae* expressing CXCL-10	Vaccines as immunotherapy	*L. major*	Mouse	(81)
Chitin and chitosan	Cytokine and immunomodulator	*L. major*	Mouse	(83)
Recombinant CP from *Leishmania infantum chagasi* and *Propionibacterium acnes*	Vaccines as immunotherapy	*L. infantum*	Dog	(88)
*L. infantum* lysate and antimony	Vaccines as chemoimmunotherapy	*L. infantum*	Dog	(94)
Leish-110f, MPL-SE, and antimony	Vaccines as chemoimmunotherapy	*Leishmania chagasi*	Dog	(95)
LEISH-F1, MPL-SE, and glucantime	Vaccines as chemoimmunotherapy	*L. infantum*	Dog	(87)
*L. major* antigen, *Mycobacterium vaccae*, and meglumine antimoniate	Vaccines as chemoimmunotherapy	*L. infantum*	Dog	(96)
Saponin-enriched Leishmune	Vaccines as immunotherapy	*L. chagasi*	Dog	(85)
Saponin-enriched Leishmune and allopurinol or allopurinol/amphotericin B	Vaccines as chemoimmunotherapy	*L. chagasi*	Dog	(86)

*Pam3Cys, tripalmytoil-cysteine; MPL-A, monophosphoryl lipid A; IL, interleukin; Ab, antibody; GITR, glucocorticoid-induced TNF receptor-related protein; SSG, sodium stibogluconate; HNP1, human neutrophil peptide-1; CXCL-10, interferon-gamma-induced protein 10; CP, cysteine protease; MPL-SE, monophosphoryl lipid A-stable emulsion; hIL-10, human monoclonal Ab*.

## Status of Field Trials

Following success of a number of vaccine candidates at laboratory evaluation, a few have been tested in the field and are listed in Table 3. Notably, Canileish, recently licensed for prophylaxis against canine VL has demonstrated Th1 biased persistent antileishmanial immunity (72, 97, 98). The success of other two licensed canine vaccines: Leish-Tec (*L. donovani* A2 protein-adenovirus) and Leishmune (FML-saponin formulation) have persuaded researchers to develop human leishmaniasis vaccine. LEISH-F1 (formerly Leish-111F), comprising of a fusion of three relatively conserved *L. major* antigens (TSA, LmStI1, and *Leishmania* elongation initiation factor) formulated as stable emulsions of MPL-A in squalene oil, is the first among defined vaccines for leishmaniasis to be clinically evaluated (99, 100). It has been reported that in animal models LEISH-F1 + MPL-SE stimulated partial protection against VL. However, Phase III trial of LEISH-F1 + MPL-SE showed unsuccessful results in defending dogs against infection (101). Human phase I trials of LEISH-F1 + MPL-SE targeting VL and CL were conducted in Colombia (2007), Brazil (2007), Peru (2007), and India (2008) demonstrating the formulation to be safe, immunogenic, and well tolerated in people irrespective of their serostatus (102). Moreover, the LEISH-F1 + MPL-SE was also found to have therapeutic significance when used with chemotherapy in patients with ML (100). The noteworthy early success of the LEISH-F1 + MPL-SE has encouraged IDRI researchers to redesign this vaccine candidate into a new construct LEISH-F2. The new candidate has a deleted N-terminal histidine tag so as to keep the recombinant molecule close to its original form with replacement of a residue Lys274, a potential site for proteolytic activity, with Gln (102). Promising phase I trial of LEISH-F2 + MPL-SE has lead to a phase II trial where the safety, immunogenicity, and efficacy were studied in evaluation with standard chemotherapy in adolescent and adults participants infected with CL [www.ClinicalTrials.gov. A study of the efficacy and safety of the LEISH-F2 + MPL-SE vaccine for treatment of CL; 2013]. A third candidate LEISH-F3 under investigation by IDRI, a tandemly fused polypeptide of open reading frame of two *Leishmania* proteins: *L. infantum/donovani* nonspecific nucleoside hydrolase protein (aa 1–314) and *L. infantum* sterol 24-c-methyltransferase protein (aa 2–353), is in a phase I trials in USA. These trials aims to assess the safety and immunogenicity of unadjuvanted LEISH-F3 with variety of adjuvants likely to be glucopyranosyl lipid A formulated as stable emulsion (GLA-SE), MPL-SE, second generation lipid adjuvant stable emulsion (SLA-SE) in different studies [www.ClinicalTrials.gov. Phase 1 LEISH-F3 vaccine trial in healthy adult volunteers; 2014, LEISH-F3 + GLA-SE and the LEISH-F3 + MPL-SE Vaccine; 2016, Phase 1 LEISH-F3 + SLA-SE Vaccine Trial in Healthy Adult Volunteers; 2016] (103).

**Table 3:** Vaccine field trials.

Description of antigen	Disease	Clinical trial phase	Vaccine type	Adjuvant	Reference
Canileish (*Leishmania infantum* ESP)	Canine visceral leishmaniasis (VL)	Licensed	Prophylactic	QA-21	(72, 97, 98)
Leish-Tec (*Leishmania donovani* A2 protein-adenovirus)	Canine VL	Licensed	Prophylactic	Saponin	(54)
Leishmune (purified *L. donovani* fraction FML)	Canine VL	Licensed	Prophylactic	Saponin	(46)
Gentamicin-attenuated *L. infantum*	Canine VL	Preclinical	Prophylactic	–	(104)
LEISH-F1 (fusion of *Leishmania major* TSA, LmStI1, and LeIF)	VL and cutaneous leishmaniasis (CL)	Phase II	Prophylactic and immunotherapeutic	MPL-SE	(99, 100)
LEISH-F2 (fusion of *L. major* TSA, LmStI1, LeIF-deleted histidine tag, and Lys274 replaced with Gln)	CL	Phase II	Prophylactic	MPL-SE	www.ClinicalTrials.gov. A study of the efficacy and safety of the LEISH-F2 + MPL-SE vaccine for treatment of CL; 2013
Leish-F3 (fusion of *L. infantum/donovani* non specific NH protein and *L. infantum* SMT)	Healthy volunteers	Phase I	Prophylactic	GLA-SE	www.ClinicalTrials.gov. Phase 1 LEISH-F3 vaccine trial in healthy adult volunteers; 2014, LEISH-F3 + GLA-SE and the LEISH-F3 + MPL-SE Vaccine; 2016, Phase 1 LEISH-F3 + SLA-SE Vaccine Trial in Healthy Adult Volunteers; 2016 (103)
ChAd63-KH [adenovirus expressing synthetic gene (KH) encoding two *Leishmania* proteins KMP11 and HASPB]	Healthy volunteers	Phase I	Prophylactic	–	(105)

*ESP, excreted-secreted proteins; FML, fructose mannose ligand; TSA, thiol-specific antioxidant; LmTSI1, L. major homolog of eukaryotic stress-inducible protein 1; LeIF, Leishmania elongation initiation factor; NH nucleoside hydrolase; SMT, sterol 24-c-methyltransferase; MPL-SE, monophosphoryl lipid A-stable emulsion; GLA-SE, glucopyranosyl lipid A; KMP11, kinetoplastid membrane protein 11; HASPB, hydrophilic acylated surface protein B*.

Other group has explored the safety, tolerability, and immunogenicity of vaccine candidate ChAd63-KH for human VL and post kala azar dermal leishmaniasis (PKDL) in Phase I clinical trials. ChAd63-KH is a simian adenoviral vaccine with defective replication, which expresses a novel synthetic gene (KH) encoding two *Leishmania* proteins kinetoplastid membrane protein 11 and hydrophilic acylated surface protein B. Phase I trial showed induced innate response characterized by activation of DCs and production of IFN-γ along with robust CD8^+^ T-cell response, which suggests the further development of ChAd63-KH as vaccine for VL and PKDL (105).

## Challenges

For the effective control and complete eradication of any infectious disease, the potential approach that can be exploited as an economical means is vaccination. Over the past two decades, immunotherapy, either alone or in combination with chemotherapy, has been developed as an additional approach to combat leishmaniasis. Lifetime immunity against reinfection manifests possibility of developing an effective vaccine (prophylactic and therapeutic) against leishmaniasis. Nevertheless, an antileishmanial vaccine for human administration is still unavailable. Therefore, the present scenario augments questions concerning the issues or limitations in the advancement of effective interventions against eradication of leishmaniasis. Some of the major challenges that need to be addressed are as follows:

1. Vaccines against leishmaniasis, malaria, schistosomiasis and a number of other bacterial and viral diseases are unappealing to the industry considering limits of financial benefits (300–800 million US dollars) (1). According to the G-Finder, over US$ 66 million has been granted for research and development of vaccine, preventative, and therapeutic, against leishmaniasis largely from chief public sector and charitable trusts (from the year 2007 to 2013). Some of the major funding sources are Carlos Slim Foundation, Bill & Melinda Gates Foundation, Wellcome Trust, Indian Council of Medical Research, European Commission, Institute Pasteur, German Federal Ministry of Education and Research (BMBF), and the U.S. National Institutes of Health (Policy Cures. G-Finder; 2015). In addition, new and enhanced funding from public–private joint ventures and pharmaceutical companies will be encouraging to boost vaccine (preventive and therapeutic) research.
2. *Leishmania-*infected individuals gain considerable lifelong immunity to reinfection, suggesting the feasibility of vaccination. However, regardless of many potential vaccine candidates, translation of these to develop a human administrable antileishmaniasis vaccine is still arduous. Selection of promising vaccine candidates has persistently been a complex issue. As reviewed in this article, a number of antigens (Table 1) have been tested with varied success based on the animal model and the vaccine formulation. The difference in opinion regarding the choice of antigens has resulted in a never-ending argument where some proclaim for a molecularly outlined formulation whereas others postulate for a live attenuated vaccine. For a human administrable vaccine and/or immunotherapy, there is an imperative need to imply a coordinated approach that minimizes the toxicity of live vaccine and maximizes the immunogenicity and efficacy of defined vaccines and immunotherapeutic agents.
3. An appealing approach to maximize the immunogenicity of the defined vaccine is the use of a suitable adjuvant (immunomodulators). The factors considerably determining the suitability of an adjuvant are the route of administration, the nature of antigens like solubility, the course of immunization, and the nature of immune response required. Development of an efficient antigen-adjuvant formulation primarily requires an in-depth knowledge of the mode of action, toxicity, and human administrability of the adjuvant. Chemically defined and licensed adjuvants that can elicit cell-mediated immune responses seem to be encouraging candidates for development of antileishmanial vaccines and immunotherapy.
4. *Leishmania* infection follows a complex clinical outcome varying from the cutaneous to visceral form as the parasite is equipped for generating an extensive assortment of atypical and uncommon variations. The virulence factors as well as in the immune responses induced by the different strains and species of *Leishmania* is not fully understood. Improved understanding of the immunobiology and vaccine (prophylactic and therapeutic) development prerequisites for the different forms of leishmaniasis will provide tools that can be exploited to overcome the virulence dynamics of *Leishmania* species.
5. Suitability of mouse and hamsters as disease model for leishmaniasis is questionable as it imitates only some facets of the leishmaniasis in human. For example, the elicited immune response and the resultant disease are governed by the choice of mouse strains, route and mode of challenge infection. The immune responses leading to protection in humans have not been fully elucidated due to lack in correlation to the immune response in the animal model. In order reduce the mismatch between laboratory disease models and human trials, genetically modified animal model expressing human leukocyte antigen (HLA) molecules in mice were generated (106), but the immune responses generated in these preliminary models were more restricted toward mouse MHC than human HLA. Replacement technique of MHC with HLA can be potentially exploited to generate optimized humanized animals for preclinical studies.

## Conclusion

Most conventional treatment option for leishmaniasis is highly expensive and toxic drugs. Vaccination (prophylactic and/or therapeutic), if possible, can be considered to be the most efficient strategy to control this infectious disease. Leishmaniasis is not an exception as patients cured of the disease become impervious to further infection. A plethora of vaccine candidates against leishmaniasis has been explored ranging from live vaccine to recombinant polyprotein and multiantigenic T-cell epitope-based vaccines. Moreover, chemotherapy along with immunotherapy that can elicit protective immune response can clear infection more effectively providing better possibility of recovery in patients. Subunit vaccine candidates—Leish-F1, Leish-F2, Leish-F3, and ChAd63-KH—are currently in different stages of clinical trials have kept alive the optimism for a licensed human vaccine (prophylactic and/or therapeutic) in near future. Although, licensed vaccines for canine leishmaniasis are available, the scope for improvement with newer approaches remains undaunted. With the rapid progress in understanding the propagation of protective immunity during leishmaniasis, development of better correlates of immunity to evaluate vaccines, novel delivery systems, immunotherapy with or without drugs, immunomodulators and adjuvant together with updated revelation in genetic information about the parasite have opened up opportunities for advanced research in the vaccine field. Moreover, if funding trust can lead the long road of vaccine development, the unmet goal of alternative approaches to chemotherapy will be achieved very soon.

## Author Contributions

ND collected literature and drafted the manuscript. MS, AS, and NA edited the manuscript. NA finalized the manuscript.

## Conflict of Interest Statement

The authors declare that the research was conducted in the absence of any commercial or financial relationships that could be construed as a potential conflict of interest.
